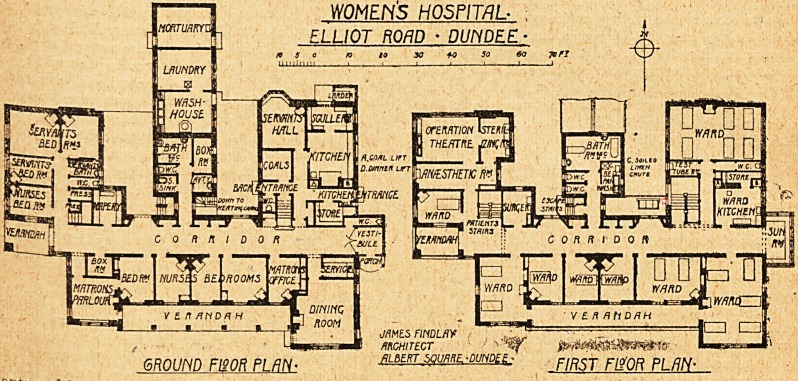# Women's Hospital, Dundee

**Published:** 1917-11-10

**Authors:** 


### HOSPITAL ARCHITECTURE AND CONSTRUCTION.

### Women's Hospital, Dundee.

THIS small hospital, which is described as "for the treatment of women in sickness," and is therefore presumably not confined to diseases special to women, was the gift of Mrs. F. B. Sharp, of Tarvit, Cupar, Fife.

The building is on two floors, the ground floor being devoted to administration and the accommodation of staff, and the upper floor to wards and operation unit.

Dealing with the upper floor first, there are eight wards, one with six beds, one with five beds, two with two beds, and the rest with one bed each. The ward kitchen, which has to serve the whole floor, is at one end of the building, instead of being in the centre of its work. At the back on the north side is a projecting block, cut off from the main corridor by a lobby, containing a bathroom with two baths, two w.c.s, and a sink room; while between the ward kitchen and the north-east ward is a w.c. with an abnormally long lobby, but no attempt at a "cut-off." Opposite is the testing room.

The operation suite comprises a theatre, sterilising room, and anæsthetic room. The theatre is distinctly small, and the anæsthetic room is so planned that patients must pass through it after being operated on. It is, moreover, very narrow and scarcely affords sufficient space for manœuvring an ambulance with ease. Further, this anæsthetic room must be traversed by everyone seeking entrance to the operating theatre or the sterilising room from the corridor; this fatal defect in planning has been many times pointed out, and has been more than once specifically dealt with in THE HOSPITAL ("The Planning of the Modern Operating Theatre Unit," July 13, 1912, p. 389; "The Anæsthetist's Ante-Room," April 30, 1910, p. 149). There does not appear to be any room provided for the surgeons to prepare themselves for operations, unless the room marked "Surgery" is intended for this purpose. If so, it is extremely badly placed.

The ground floor contains the usual domestic offices, four bedrooms for nurses, an office, sitting room and bedroom for matron, three servants' bedrooms, a dining room which presumably serves also as sitting room for nurses, and a one-storey wing in the centre which contains washhouse, laundry, and mortuary. Bathroom for nurses and servants, w.c.s, and sink rooms are provided, also a very small boxroom lighted by a borrowed light, and a linen store.

The staircase marked "Patients" must surely be dark, the only window being quite a small one on the opposite side of the corridor, and even this appears to be screened off on the upper floor.

We cannot but regret that a little more intelligent knowledge of the requirements of hospital planning was not brought to bear in the designing of this building.

The architect was Mr. James Findlay, of Dundee.

## Figures and Tables

**Figure f1:**